# Preliminary experience of performing a video endoscopic inguinal lymphadenectomy using a hypogastric subcutaneous approach in patients with vulvar cancer

**DOI:** 10.3892/ol.2014.2757

**Published:** 2014-12-02

**Authors:** HE WANG, LI LI, DESHENG YAO, FEI LI, JIEQING ZHANG, ZHIJUN YANG

**Affiliations:** Department of Gynecologic Oncology, Affiliated Tumor Hospital of Guangxi Medical University, Nanning, Guangxi 530021, P.R. China

**Keywords:** vulvar cancer, endoscopy, inguinal lymphadenectomy

## Abstract

To evaluate the feasibility and surgical outcome of video endoscopic inguinal lymphadenectomy (VEIL) using a hypogastric subcutaneous approach, 21 patients with vulvar cancer who underwent this procedure were included in the present study. Between December 2010 and March 2013, 21 consecutive patients with vulvar cancer underwent radical vulvectomy and VEIL using a hypogastric subcutaneous approach. The intraoperative and post-operative results and follow-up data were retrospectively analyzed. No intraoperative complications occurred. The mean duration of surgery for the endoscopic inguinal lymphadenectomies was 130 min (range, 80–180 min), with a mean estimated blood loss of 103 ml (range, 30–350 ml). The mean lymph node yield was 15 (range, 10–22 lymph nodes). The suction drains were removed after a mean duration of 7 days (range, 5–11 days). No skin-related complications were observed in the groin region and a lymphocele was only observed in 1/21 (4.8%) patients. After a mean follow-up period of 17 months (range, 3–31 months), recurrence was found in only one patient. All the patients were alive at the time of publication. Based on our preliminary experience, performing VEIL using a hypogastric subcutaneous approach is a safe and feasible technique for patients with vulvar cancer. These results indicate that this surgical technique may decrease the post-operative morbidity of lymphadenectomy without compromising the therapeutic efficacy. Future prospective studies with a greater sample size and a longer duration of follow-up are required.

## Introduction

Vulvar cancer is a rare disease that accounts for 3–5% of all female genital malignancies (1). Lymphatic metastasis is one of the primary modes by which vulvar cancer spreads, and the inguinal lymph nodes are the first affected sites (2). Thus, despite the positive outcome of the sentinel lymph node procedure (3–5), radical vulvectomy and bilateral inguinal lymphadenectomy remain the first choice of treatment for invasive vulvar cancer with the risk of regional metastasis. Inguinal lymphadenectomy cannot only remove all high-risk lymph nodes, it can also provide evidence for subsequent radiation therapy and predict the survival of vulvar patients by determining their nodal status (6). However, the conventional open method of inguinal lymphadenectomy may result in numerous wound-related and lymphatic complications (7). Video endoscopic inguinal lymphadenectomy (VEIL) using the limb subcutaneous approach has recently been reported as a potentially less invasive alternative to open inguinal lymphadenectomy, in order to decrease complications following inguinal lymphadenectomy for patients with certain genitourinary malignancies (8–11). The concept of VEIL was first described by Dargent *et al* (12) in 1996 and since then, certain technical changes have been introduced. In the limbs, the triangular working space may be obtained by the introduction of the laparoscopic instruments via three (8,9,11) or a single (10) working port. However, if the obtained frozen sections reveal deep inguinal nodes that are positive for metastasis, a laparoscopic pelvic lymphadenectomy must be performed with three or more abdominal working ports. To avoid the requirement to perform surgery via the limb and the abdomen, a hypogastric subcutaneous approach may result in the use of less access routes, leading to a possible reduction in morbidity from the breakdown of the wounds caused.

## Materials and methods

### Patients

Between December 2010 and March 2013, 21 consecutive patients with invasive vulvar cancer underwent radical vulvectomy and standard inguinal lymphadenectomy by endoscopy. Prior to surgery, vulvar cancer was confirmed in these patients via lump biopsy. The mean age of the patients was 59.3 years (range, 15–74 years). Written informed consent was obtained from all patients and the study was approved by the ethcs committee of the Affiliated Tumor Hospital of Guangxi Medical University (Nanning, China).

### VEIL

All procedures were performed with the patient under general endotracheal anesthesia. The technique that is normally used for a standard inguinal lymph node dissection was used in the present study patients. The aim of this approach was to remove all of the lymph nodes that were in the most probable location for first-line lymphatic invasion. The technique used for the selection of the incision sites and the creation of space was based on the technique described by Xu *et al* (13).

### Patient positioning, placement of trocars and development of working space

The patient was positioned in a supine position with slight external rotation of the two limbs to provide adequate exposure to the inguinal region and perineum. The first 10-mm umbilical trocar was placed at the umbilicus and passed subcutaneously downward to the inguinal region. Subsequent to completing the laparoscopic instrument insertion, the maximum insufflation pressure was initially set at 15 mmHg, and carbon dioxide was infused to create a working space. After the working space was established, the insufflation pressure was reduced to 8–10 mmHg to prevent widespread subcutaneous emphysema. Subsequently, the second 5-mm trocar was inserted under direct endoscopic vision (A50022A; Olympus Corporation, Tokyo, Japan) into the subcutaneous space at the midpoint between the umbilicus and pubic symphysis, and two additional 5-mm ports were inserted into the subcutaneous space at double McBurney’s point. Under direct vision, the working space was developed between the subcutaneous fibrofatty packet of the anterior abdominal region and Camper’s fascia by ultrasonic Harmonic scalpel (Johnson & Johnson, Shanghai, China). The dissection was performed ~5 cm superior to the inguinal ligament and deep to the Camper’s fascia, and was extended up to the external oblique muscle aponeurosis. The fibrofatty packet containing the lymph nodes was dissected from the top of the incision down to the inguinal ligament.

### Dissection and identification of the femoral triangle landmarks

**Figure 1:**
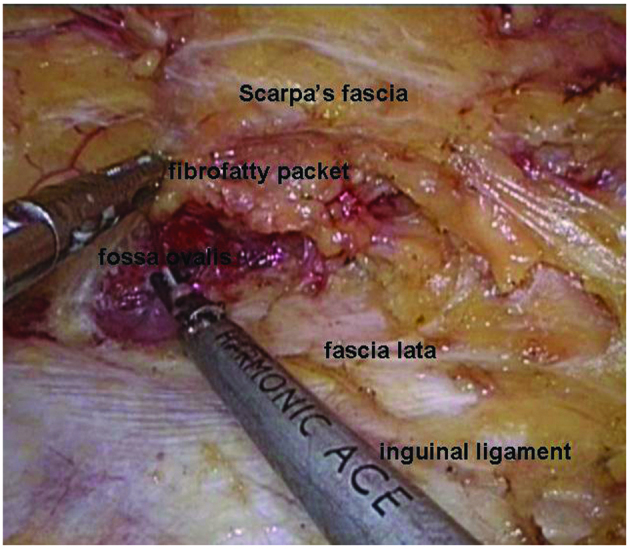
Dissection and separation of the fibrofatty packet below the inguinal ligament and exposure of the fascia lata and the fossa ovalis.

The main landmarks of dissection were the superior to inguinal ligaments, which is inferior to the apex of the femoral triangle, medial to the adductor longus muscle and lateral to the sartorius muscle. Following dissection and separation of the fibrofatty packet below the inguinal ligament using an ultrasonic scalpel, the fascia lata was exposed (Fig. 1). Subsequently, the dissection proceeded downward along the surface and ran closely along the fascia lata to 3 cm below the pubic tubercle. When the fascia lata was completely separated, the femoral triangle was exposed with the saphenous vein, sartorius muscle and adductor longus muscle.

### Dissection of saphenous vein and its branches and excision of the deep inguinal nodes

**Figure 2:**
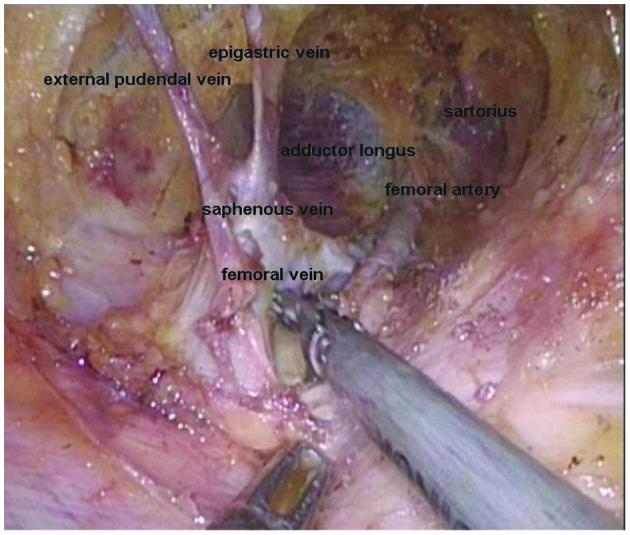
Dissection and separation of the saphenofemoral junction to expose the saphenous vein and femoral artery and vein branches. The main saphenous vein was conserved and the lymph nodes were removed on each side of the vein and surrounding the fossa ovalis to the apex of the femoral triangle.

When the superior edge of the saphenous vein was exposed where it entered the femoral vein at the fossa ovalis, the entire saphenofemoral junction and all of its superficial branches were dissected, including the superficial epigastric, superficial iliac circumflex and superficial external pudendal veins, while preserving them as much as possible. The main saphenous vein was conserved and the lymph nodes were removed on each side of the vein and surrounding the fossa ovalis to the apex of the femoral triangle. Subsequently, the femoral artery and vein in the superior to inferior direction were dissected within the femoral triangle (Fig. 2). All the lymph nodes superior to and below the saphenofemoral junction, as well as the deep inguinal nodes (Cloquet’s nodes) medial to the femoral vein up to the inguinal ligament, were removed.

### Removal of specimen and drain placement

When the packet consisting of the fibrofatty tissue and lymph nodes was dissected at the saphenofemoral junction, it was completely liberated. The packet was then placed in an endoscopic specimen retrieval bag and withdrawn from the apical port of the femoral triangle. The port incisions were closed following the placement of a fully fluted pediatric stomach tube via the port of the apex of the femoral triangle, and the drain was connected to a continuous vacuum suction device. The post-operative surgical field was oppressed with a saline soft bag. The same procedure was performed on the other side for inguinal lymph node dissection.

### Pelvic lymphadenectomy and radical vulvectomy

Pelvic lymphadenectomy was performed when the inguinal node frozen sections were deemed positive for metastasis. Without changing the position of the ports, pelvic lymph node resection was performed, as previously described in patients with cervical cancer (14). A modified radical vulvectomy was performed (15).

## Results

The clinical and pathological characteristics of the 21 patients are shown in Table I. The mean diameter of the primary tumor was 3.4 cm (range, 1.0–7.0 cm). According to the International Federation of Gynecology and Obstetrics, 2009 classification (16), 11 of the 21 patients exhibited stage Ib disease, four exhibited stage II disease, five exhibited stage III disease and one exhibited stage IV disease. A total of 18 patients were diagnosed with squamous cell carcinoma, two with sarcoma and one with a primitive neuroectodermal tumor.

The intraoperative and post-operative periods were uneventful for all patients (Table II). No intraoperative complications were observed and all the procedures were performed successfully. The mean duration of surgery was 130 min (range, 80–180 min) and the mean number of lymph nodes removed was 15 (range, 10–22). The mean estimated blood loss was 103 ml (range, 30–350 ml). Histological analysis confirmed inguinal lymph nodes that were positive for metastasis in six patients and thus, additional pelvic inguinal lymph node dissections were performed. The suction drains were removed after a mean period of seven days (range, 5–11 days) following surgery. No skin-related complications were observed in the groin region. One lymph-related complication was observed in in the form of a lymphocele in 1/21 patients (4.8%). Six patients with positive inguinal lymph nodes received regional radiation therapy at a total dose of 45–50 Gy. The other patients required no adjuvant therapy. The mean duration of follow up was 17 months (range, 3–31 months), and cancer recurrence was only identified in one patient, which was verified by biopsy. The patient subsequently underwent four cycles of Taxol + cisplatin chemotherapy, with a routine dosage of 175 mg/m^2^ Taxol and 70 mg/m^2^ cisplatin over the course of three weeks. Currently, no patients have succumbed and no other tumor recurrence has occurred.

## Discussion

Due to the local invasion and multicentric occurrence of vulvar cancer, groin node metastases in patients with vulvar cancer occur in 9–40% of cases (17,18). Open inguinal lymphadenectomy is an essential conventional management technique, but the associated morbidity is high. VEIL using the limb subcutaneous approach is a novel, minimally invasive technique that was first reported by Machado *et al* (19) in 2005, for use in penile cancer patients. The aim of VEIL is to replicate the standard radical procedure, but with decreased morbidity. VEIL using this approach has subsequently been performed in a number of studies (20–23), and the results have demonstrated similar node counts to the standard procedure, but with decreased complications.

Although penile and vulvar cancers are treated using the same inguinal lymphadenectomy procedure, only a small number of studies have investigated the use of VEIL for vulvar cancer patients (13,24). All working ports for the VEIL procedure in penile cancer are located in the lower limbs, however, laparoscopic pelvic lymphadenectomy must be performed in the vulva in cancer patients with positive groin nodes, thus, the lower limb ports used for penile cancer patients are not applicable for vulvar cancer patients. Additional abdominal incisions are therefore essential, which increases the degree of surgical trauma. In this study, the port placement was modified to the hypogastrium, in accordance with a study performed by Xu *et al* (13). In comparison to the reported VEIL technique using the limb subcutaneous approach with six ports in the two lower limbs, the VEIL technique using the hypogastric subcutaneous approach with four ports, which was used in the present study, has the advantage of being less invasive due to fewer incisions. There is also a reduced rate of post-operative lower limb movement disorder, and it is easier to convert the procedure to a laparoscopic pelvic lymphadenectomy, without additional trocar insertion, in cases where inguinal lymph node metastasis is confirmed during surgery. This technique includes the superficial and deep inguinal lymphadenectomy and has now been performed successfully in 21 patients. Although the mean duration of surgery (130 min) is marginally longer than the standard procedure, the mean estimated blood loss (103 ml) and the mean number of nodes removed (15 lymph nodes) is similar to that reported in the literature for the standard procedure (13,20–23). Therefore, the VEIL-hypogastric technique is recommended for future use rather than the VEIL-limb and open inguinal lymphadenectomy techniques in those patients at a higher risk of inguinal or pelvic metastases.

Uncertainty may exist with regard to the potential risk of tumor seeding due to infra-umbilical emphysema. Although no studies have been performed that have analyzed the risk of tumor seeding when using video endoscopic inguinal lymph node dissection, certain studies have evaluated the role of a pneumoperitoneum in tumor seeding in laparoscopic surgeries (25,26). In previous animal and human studies, CO_2_ has been shown to be unable to aerosolize large quantities of tumor cells using pressures of 8–15 mmHg (26,27). Therefore, in the present study, the maximum insufflation pressure was initially set at 15 mmHg, and carbon dioxide was infused to create a working space. Once the working space had been created, the insufflation pressure was reduced to 8–10 mmHg to prevent widespread subcutaneous emphysema.

As VEIL is carried out in a small space, a clear view of the inguinal anatomy is key for a successful surgery. VEIL may only be performed by gynecological oncology physicians who simultaneously master the open method of inguinal lymphadenectomy and the skills of endoscopy. Dissection of the groin lymph nodes should run deeply to the external oblique muscle aponeurosis and fascia lata, and shallow to the Camper’s fascia and skin using a Harmonic scalpel. In the present study, the fibrofatty packet containing the lymph nodes was dissected from the top down to the inguinal ligament. Below the inguinal ligament, the surgeon continued to dissect and separate the nodal packet to expose the fascia lata and fossa ovalis. Subsequently, the dissection proceeded downward on the surface and was run close to the fascia lata to 3 cm below the pubic tubercle. When the fascia lata is absolutely separated and incised, the saphenous hiatus and the femoral triangle should be completely revealed (13,28). Exposing the saphenofemoral junction exposes the deep inguinal nodes (Cloquet’s nodes) located medial to the femoral vein up to the inguinal ligament (13,24). The lymph node resection is performed superior to and below the saphenofemoral junction and medial to the femoral vein, without removing the cribriform fascia or dissecting lateral to the femoral artery, and below to the fossa ovalis. Using this technique, the number of nodes that are obtained is almost identical to that obtained with open groin node dissection.

Additionally, the skin must not be cut through in the process of separating the subcutaneous fibrofatty packet. Intraoperatively, electrocoagulation must be used as much as possible, and the drain should be placed through the port of the apex of the femoral triangle and connected to a continuous vacuum suction device to reduce the development of a lymphocele. Post-operatively, the operative field should be oppressed with a saline soft bag (29), and the drain should not be removed until the drainage totals <20 ml/day. In the present study, no skin-related complications were observed in the groin region. A lymphocele was observed in 1/21 patients (4.8%). Therefore, this technique should aid in the reduction of morbidity compared with the use of classic inguinal lymphadenectomy.

Regarding the long-term outcome for the patients who undergo endoscopic inguinal lymphadenectomy, in a study by Tobias-Machado *et al* (9) there were no recurrences detected during a mean follow-up time of 31.9 months, and the number of lymph nodes was similar to that using the conventional open surgery. In the present study, the patients underwent a mean follow-up period of 17 months, and the recurrence of cancer was found in just one patient who was subsequently treated with chemotherapy. Currently, no patients have succumbed and no other tumor recurrence has occurred. VEIL can therefore be considered as a promising minimally invasive approach for radical inguinal dissection in patients with vulvar cancer.

The preliminary results of the present study show that performing VEIL using the hypogastric subcutaneous approach is safe and feasible in patients with vulvar cancer. The procedure can obtain similar therapeutic efficacy to the conventional surgery, but with reduced surgical morbidity. The technique is a promising, minimally invasive alternative surgical treatment for vulvar cancer, however, long-term studies with a greater number of patients are required.

## Figures and Tables

**Table I:** Clinical and pathological characteristics of 21 vulvar cancer patients.

Characteristic	Value
Age, years	
Median	59.3
Range	15.0–74.0
Diameter of primary tumor, cm	
Median	3.4
Range	1.0–7.0
Clinical stage of primary tumor^a^, n (%)	
Ib	11 (52.4)
II	4 (19.0)
III	5 (23.8)
IV	1 (4.8)
Pathological type of primary tumor, n (%)	
Squamous cell cancer	18 (85.7)
Sarcoma	2 (9.5)
Primitive neuroectodermal tumor	1 (4.8)

a International Federation of Gynecology and Obstetrics staging, 2009.

**Table II:** Intraoperative and post-operative data of 21 vulvar cancer patients.

Parameter	Value
Surgical time, min	
Median	130
Range	80–180
Operative blood loss, ml	
Median	103
Range	30–350
Node count, n	
Median	15
Range	10–22
Lymph node metastasis, n (%)	
Negative	15 (71.4)
Positive	6 (28.6)
Duration of drain, days	
Median	7
Range	5–11
Complications, n (%)	
Inguinal wound necrosis	0 (0.0)
Lymphocele	1 (4.8)
